# Innovative Strategies for Early Autism Diagnosis: Active Learning and Domain Adaptation Optimization

**DOI:** 10.3390/diagnostics14060629

**Published:** 2024-03-16

**Authors:** Mohammad Shafiul Alam, Elfatih A. A. Elsheikh, F. M. Suliman, Muhammad Mahbubur Rashid, Ahmed Rimaz Faizabadi

**Affiliations:** 1 Department of Mechatronics Engineering, International Islamic University Malaysia, Jln Gombak, Kuala Lumpur 53100, Malaysia; alam.s@live.iium.edu.my (M.S.A.); amed.rimaz@live.iium.edu.my (A.R.F.); 2 Department of Electrical Engineering, College of Engineering, King Khalid University, Abha 61421, Saudi Arabia; eelsheikh@kku.edu.sa (E.A.A.E.); fmsuliman@kku.edu.sa (F.M.S.)

**Keywords:** ASD, deep learning, facial images, active learning, domain adaptation

## Abstract

The early diagnosis of autism spectrum disorder (ASD) encounters challenges stemming from domain variations in facial image datasets. This study investigates the potential of active learning, particularly uncertainty-based sampling, for domain adaptation in early ASD diagnosis. Our focus is on improving model performance across diverse data sources. Utilizing the Kaggle ASD and YTUIA datasets, we meticulously analyze domain variations and assess transfer learning and active learning methodologies. Two state-of-the-art convolutional neural networks, Xception and ResNet50V2, pretrained on distinct datasets, demonstrate noteworthy accuracies of 95% on Kaggle ASD and 96% on YTUIA, respectively. However, combining datasets results in a modest decline in average accuracy, underscoring the necessity for effective domain adaptation techniques. We employ uncertainty-based active learning to address this, which significantly mitigates the accuracy drop. Xception and ResNet50V2 achieve 80% and 79% accuracy when pretrained on Kaggle ASD and applying active learning on YTUIA, respectively. Our findings highlight the efficacy of uncertainty-based active learning for domain adaptation, showcasing its potential to enhance accuracy and reduce annotation needs in early ASD diagnosis. This study contributes to the growing body of literature on ASD diagnosis methodologies. Future research should delve deeper into refining active learning strategies, ultimately paving the way for more robust and efficient ASD detection tools across diverse datasets.

## 1. Introduction

Autism spectrum disorder (ASD) is a neurodevelopmental condition distinguished by repetitive patterns of behavior, interests, or activities and difficulties in social communication [1]. People with ASD may experience a wide range of challenges, which can affect their symptoms and functional abilities. Diagnosing ASD requires a discerning eye for its multifaceted nature, and the limitations of traditional methods often obscure a clear picture [2]. Nevertheless, the urgency is undeniable, as ASD affects one in every hundred infants worldwide, according to the World Health Organization [3]. Without definitive biomarkers, timely detection is critical for effective intervention and support [4]. The timely identification of ASD enables the implementation of customized interventions throughout the developmental phases, which may positively impact the long-term prognosis of those affected [5].

Conventional diagnostic approaches for ASD regarded as golden standards often lean heavily on behavioral assessments and expert interviews. While these methods offer state-of-the-art diagnoses, their inherent subjectivity and human bias highlight the need for more objective and effective tools [6]. This is where deep learning steps in, poised to revolutionize the field of ASD diagnosis. Deep learning’s ability to analyze intricate patterns and data representations holds immense promise for automating the diagnostic process [7]. For example, the utilization of deep learning algorithms on neuroimaging data, such as functional magnetic resonance imaging (fMRI) [8] and electroencephalography (EEG) [9], has facilitated the identification of neurological variances linked to autism. While this method enables precise early predictions of ASD, it requires substantial costs and the expertise of specialized medical professionals to obtain neuroimaging data. Utilizing facial images for ASD diagnosis offers a straightforward and user-friendly approach for quick initial screening, eliminating the need for expert intervention or expensive diagnostic tests. Facial image datasets have demonstrated considerable potential as a valuable resource across various applications, most notably within the domain of deep learning [10]. These datasets offer compelling advantages, are readily available, and are primed for deep learning algorithms. Moreover, facial images possess inherent characteristics that make them efficient training tools for applications like facial recognition and emotion analysis, ultimately aiding in developing accurate ASD diagnostic models [11].

While traditional methods offer valuable insights into ASD, their subjectivity and limitations highlight the need for objective, automated tools for early detection. This is where active learning, a machine learning paradigm that prioritizes informative data for annotation, emerges as a powerful tool with proven success in diverse fields like image classification and natural language processing [12]. Its potential in ASD diagnosis is multifaceted. Actively selecting subtle facial expressions or behavioral patterns suggestive of ASD can lead to earlier and more accurate diagnoses, potentially unlocking a crucial window for customized interventions during critical developmental phases [13]. Moreover, by prioritizing samples reflecting individual differences in ASD presentation, active learning can pave the way for personalized interventions tailored to specific needs. Furthermore, the burden of data labeling, often a bottleneck in medical image analysis, can be significantly reduced by actively choosing informative samples for annotation, improving model performance with fewer labeled examples. Finally, active learning can address the challenge of data variability across different clinical settings or populations by selecting samples that bridge the gap between domains, thereby enhancing model generalizability and robustness. This research aims to bridge the gap in ASD diagnosis by developing an ASD-specific active learning strategy, evaluating its effectiveness with diverse datasets, investigating its impact on intervention development, and collaborating with clinicians and researchers to ensure its practical and ethical implementation. By harnessing the power of active learning, we can move toward more robust, personalized diagnostic tools for ASD, ultimately improving the lives of individuals and families affected by this condition.

This study delves into domain adaptation, a crucial medical image data analysis element. The inherent diversity of imaging conditions, sources, demographics, and modalities often challenges building generalizable and robust models [14]. This diversity often stymies the development of generalizable and robust models, a challenge encountered in various medical applications beyond ASD diagnosis. For instance, accurately classifying diseases across different imaging modalities like X-rays and MRIs, personalizing drug discovery by leveraging knowledge from existing targets, or adapting image segmentation models from one anatomical region to another all benefit from robust domain adaptation strategies. Traditional approaches require retraining with completely new datasets from different domains, leading to additional labeling, preprocessing, and time constraints. Domain adaptation offers a more efficient solution by enhancing the model’s ability to generalize across domains, ensuring its efficacy in diverse clinical settings, and boosting its robustness and flexibility for a broader range of patient populations. This translates to potentially improving disease classification accuracy across modalities, accelerating personalized medicine by tailoring models to individual profiles, and streamlining medical image analysis tasks with reduced data labeling needs. Active learning plays a pivotal role in achieving this objective. Its targeted annotation and labeling of informative samples, combined with the powerful feature learning capabilities of deep learning, unlock the intricate nature of smooth adaptation across various medical image datasets [15]. By prioritizing the most pertinent data points, active learning reduces the need for extensive relabeling, significantly streamlines the model’s adaptation process, and paves the way for more efficient and adaptable diagnostic tools.

Recent studies have illuminated the potential of deep learning and facial analysis for ASD detection, yielding promising advancements. Table 1 showcases a selection of recent studies employing various convolutional neural network (CNN) algorithms and facial image datasets for ASD detection. While recent studies like those by [16,17]) using deep learning and facial analysis show promise for ASD detection, achieving accuracies above 90% on specific datasets, they all share a key limitation: They rely on single-domain data. This highlights the need for future research to explore domain adaptation strategies, ensuring models can generalize and adapt to clinical scenarios with diverse patients and imaging conditions.

Domain adaptation proves indispensable when harnessing facial images and deep learning for ASD diagnosis, owing to several compelling reasons. Primarily, the existence of diverse environments from which test case image samples originate underscores the crucial need for models to dynamically adjust to and accommodate these unique circumstances [21]. Given the impracticality of continuously retraining the model to account for each distinct modality or population, domain adaptation becomes a pragmatic alternative. To fortify the model’s robustness, identified as a critical determinant in this study, active learning is employed to retrain the models with predefined weights from the training sample of the previous domain. The significant contributions of this paper are as follows:

(a) **The assimilation of novel facial image dataset, YTUIA:** Presented as a diagnostic tool for ASD, the YTUIA dataset introduces a novel and distinctive set of features. Unlike existing datasets, YTUIA encapsulates a broader spectrum of facial expressions, ages, and ethnicities, making it a valuable addition to the landscape of ASD research. This dataset addresses a critical gap in the existing literature, where previous studies predominantly relied on single-domain data.
(b) **The optimization of a pretrained model using data from various domains:** Through the incorporation of active learning, this study pioneers an approach to optimize pretrained models using data from diverse domains. Leveraging facial images from both Kaggle ASD and YTUIA datasets, the models are fine-tuned to capture nuanced patterns associated with ASD across different populations and imaging conditions. This innovative method significantly enhances the adaptability of the model, allowing it to generalize effectively in clinical scenarios with varying patient demographics.
(c) **Enhanced weight optimization through active learning:** Active learning plays a pivotal role in the optimization process by guiding the selection of informative samples from each domain. These samples, strategically chosen based on uncertainty, refine the model’s weights, thereby improving its diagnostic accuracy. This approach mitigates the challenges posed by domain variations and ensures that the model adapts dynamically to the intricacies of each dataset. The result is a more robust and flexible diagnostic tool that excels in recognizing ASD-indicative patterns.

This multifaceted contribution, encompassing novel dataset assimilation, cross-domain optimization, and active learning-driven weight refinement, collectively advances the field of ASD diagnosis. It expands the scope of available datasets and introduces a methodology that addresses the critical need for models to adapt seamlessly to diverse clinical scenarios, ultimately paving the way for more effective and adaptable diagnostic tools in ASD research.

The initial section of the study provides an introduction, explores contemporary research, identifies gaps, and outlines potential contributions. Section 2 delves into the methodology for ASD diagnosis, detailing the newly synthesized facial image dataset, the application of active transfer learning, and the accomplished domain adaptation through active learning techniques. Subsequently, Section 3 involves the assessment of various performance indices derived from deep CNN models’ training and testing stages, emphasizing domain adoption for more accurate predictions across multiple domains. In Section 4, we discuss relevant works and explain evaluation metrics, while Section 5 concludes by providing an overview of potential future research directions and summarizing the study’s key findings.

## 2. Materials and Methods

In this study, we used an innovative approach that goes beyond conventional methods by incorporating cutting-edge techniques to substantially enhance the early identification of ASD. The Methods section provides a thorough investigation, beginning with a comprehensive overview of the active learning strategies employed to carefully choose informative facial image samples. This section delves into the complexities of domain adaptation, demonstrating how this specialized method is carefully crafted to enhance the model performance in various situations. Our methodology’s objective was to fully exploit the facial image dataset’s capabilities. Moreover, the reason for the deliberate deployment of sophisticated algorithms and adaptive learning approaches was to enhance the precision and effectiveness of early-stage ASD detection.

### 2.1. Dataset

#### 2.1.1. Dataset 1 (Kaggle)

The first dataset, defined as D1, was constructed using the facial images of autistic children and is readily accessible on the Kaggle repository [22]. The dataset comprised 2D RGB images and spans the age range of 2 to 14, mainly focusing on children aged 2 to 8 years old. The dataset had an approximate 3:1 male-to-female ratio. In contrast, the ratio for the autistic and normal control (NC) groups remained close to 1:1. The dataset consisted of train, test, and validation sets with respective proportions of 86.38%, 10.22%, and 3.41%. In each set, it was ensured that the ratio between ASD and NC classes remained constant at 1:1. The dataset, compiled by Gerry Piosenka from online sources, was devoid of demographic information such as socioeconomic status, ethnicity, clinical history, or ASD severity.

#### 2.1.2. Dataset 2 (YTUIA)

**Figure 1:**
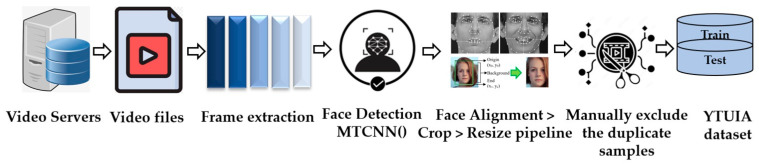
The curation process of the YTUIA dataset involved the transformation of video data into facial images.

The second facial image dataset, referred to as D2 (YTUIA-YouTube data curated in Universiti Islam Antarabangsa), comprised 75 videos extracted from the Self-Stimulatory Behaviours Dataset (SSBD), which is a well-known dataset on autism [23]. While only fifty videos are accessible on YouTube, an additional fifty were identified from therapists or specialized institutions, resulting in a selection of 100 videos from YouTube for frame extraction. As normal control samples, YouTube videos of kindergarten school activities were selected within the age category. During the preliminary stage, facial detection was performed on every frame utilizing the MTCNN algorithm. Subsequently, a meticulous preprocessing pipeline was executed, which included tasks such as aligning, cropping, and resizing as shown in Figure 1. The normal control group included a unique group of 173 individuals, 117 of whom were male, and 56 were female. The age distribution of these participants spanned from 1 to 11 years. The dataset consisted of 123 individuals in the “ASD” category, 93 of whom were male, and 30 were female. The ages of the participants ranged from 3 to 11 years. A training set with 1068 samples and a test set containing 100 samples were utilized to maintain a 1:1 ratio of individuals with ASD to NC.

### 2.2. Active Learning

Active learning has emerged as a powerful paradigm in medical image classification, offering a dynamic solution to achieve high model performance despite limited labeled data. The surge of deep learning has propelled active learning to new heights as researchers leverage the deep architectures of CNNs for robust feature extraction and classification [24]. The research in recent years has focused on overcoming challenges like scalability, computational efficiency, and adapting to diverse medical imaging modalities. This has given rise to prominent techniques like ensemble-based active learning and uncertainty sampling [25]. Studies conducted using a digital database for screening mammography show that the active learning (AL) technique can effectively reduce the cost of labeling mammographic images without compromising the accuracy of the final classification system [26]. The general flow of active learning for medical image classification unfolds in these key steps:

I. Initial Model Training: A small subset of labeled images is used to train an initial classification model.
II. Uncertainty Sampling: The model then analyzes unlabeled images, identifying those with the highest uncertainty or disagreement about their predicted class. These “informative” images are prioritized for manual annotation by expert clinicians.
III. Model Retraining: The newly labeled images are incorporated into the training set, allowing the model to refine its decision boundaries and learn from the expert annotations.
IV. Iterative Process: Steps 2 and 3 are repeated iteratively, with the model progressively improving its accuracy and confidence as it acquires more informative data points.

For the binary classification, where there are only two classes, 0 and 1, the model’s prediction $x_{k}$ is given as follows [11]:

(1) $$P_{k}=P\left(y=1|x_{k}\right)$$

Later, the most uncertain or informative images, where the model is least confident about its predictions, are identified.

(2) $$C_{least}(x_{k})=\min(P_{k},1-P_{k})$$

Lastly, the classification model is again retrained using the newly labeled data, incorporating it into the existing labeled dataset. The updated model parameter *θ* can be obtained from Bayesian inference:

(3) $$P\left(\theta \right|D_{l},D_{U})=P\left(\theta \right|D_{l},D_{U}|\theta )P(\theta )$$

where $D_{l}$ is the labeled dataset, and $D_{U}$ is the unlabeled one. We must repeat this process using the updated model to select the next set of uncertain images for manual annotation. Lastly, the most uncertain samples are identified, and experts manually annotate the selected images with their correct labels. For samples $x_{k}$

(4) $${Sample}_{uncertain}=\arg\left\{{min}_{x_{k}}C_{least}\left(x_{k}\right)\right\},k=1,2,\ldots ,N$$

Active learning effectively guides the model’s learning process by iteratively selecting and annotating the most informative images, enabling it to achieve superior performance with significantly fewer labeled data than traditional approaches.

In what follows, the sequential progression of active learning methods within the domain of medical image classification is explained shown in Algorithm 1 to clarify the progression of approaches, the mathematical underpinnings of these methods, and their effect on improving the precision of diagnoses.
**Algorithm 1** Algorithm to apply active learning for ASD screeningInput: N = Total number of samples in D2 T2 = Test set for evaluation of matrices l = Number of labeled samples on first iteration U = N − l samples, pool of unlabeled data m = Number of iterations n = $\frac{N-l}{m}$, Number of labeled samples added per iteration Start **for**
*iteration in range* (*m*) **do**  n_labeled ← l + m × n  model_train (n_labeled)  f_12_ ← feature learning (model)  w_12_ ← model_get_weights ()  prediction ← model_predict (U)  confidence ← assign_confidence (prediction)  uncertain samples ← query_on (prediction)  M_12_
← model_evaluate(T2) End

### 2.3. Domain Adaptation

**Figure 2:**
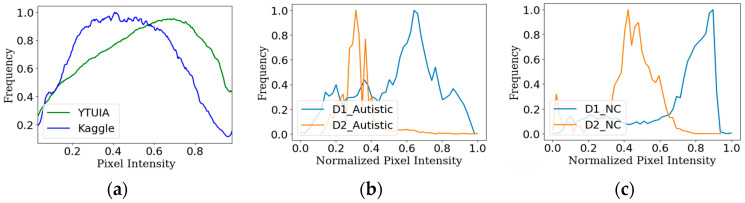
The normalized intensity distribution of (**a**) all samples of D1 and D2, (**b**) single autistic samples belonging to D1 and D2, and (**c**) single NC samples belonging to D1 and D2.

Active learning has emerged as a powerful tool for domain adaptation to expedite early autism detection through facial image analysis. This technique empowers deep learning models to extract knowledge from diverse datasets, overcoming the hurdles posed by domain shifts. Domain adaptation tackles the challenge of applying a model trained on one data source (source domain) to a different data source (target domain) with distinct characteristics. These differences can arise from various factors, such as imaging equipment variations and facial image settings. Transductive transfer learning comes into play to bridge this gap and ensure consistent performance across domains. This approach facilitates seamless knowledge transfer between models, paving the way for effective feature space adaptation [15]. Suppose

(5) $${min}_{\theta }L_{s}\left(\theta X_{S}\right)=\lambda ℋ(X_{S},X_{T})$$

where *θ* is the model update parameter, $L_{s}$ is the standard supervised loss for source domain, ℋ($X_{S}$,$X_{T}$) is the divergence between the source and target domain, and λ is the trade-off parameter between supervised loss and distribution discrepancy. The datasets utilized in this study are designated as D1 and D2, with corresponding test sets T1 and T2, as described in the preceding section. As illustrated in Figure 2a, the normalized intensity distribution of both datasets was obtained through the conversion of the pixel intensities of the facial images comprising the datasets. The discrepancy in intensity distribution corresponds to a difference in the domain [27] and, as a result, causes classification task perplexity for models. Furthermore, the T-statistic [28] yields a *p*-value of 0.15 and a T-value of 3.10, which firmly illustrates the distinction between the overall populations of D1 and D2. Moreover, Figure 2b,c illustrate the disparity in pixel intensity among sample images from the same class but obtained from separate datasets. This highlights the model’s challenges when learning features from various domain samples.

### 2.4. Convolutional Neural Networks

Deep learning has revolutionized image classification tasks, including facial recognition. Convolutional neural networks (CNNs) have emerged as powerful tools, leveraging advancements in computing power, vast training datasets, and transfer learning techniques [29,30]. In the context of autism detection, the goal is to accurately identify subtle facial features that differentiate autistic individuals from neurotypical controls (NCs). This is achieved by extracting and analyzing feature vectors from facial images using pretrained CNN models and transfer learning [31]. One can utilize a machine learning technique to perform this task by modifying the top layers of pretrained models to accommodate the necessary adjustments. The primary convolutional layers of CNN-based models, previously trained using the ImageNet dataset, extracted distinctive features of autistic and NC faces. Subsequently, the classification layers were fine-tuned specifically for binary classification. In the present study, we employed three deep CNN models—MobileNetV2, ResNet50V2, and Xception—chosen for their proven performance in image classification tasks and potential ability to capture the relevant features for autism detection. Originally trained on large datasets like ImageNet, these models were fine-tuned with autism-specific data by modifying the final layers for binary classification. This targeted approach allowed us to leverage the powerful feature extraction capabilities of pretrained models while adapting them to the specific needs of autism diagnosis [32].

#### 2.4.1. MobileNetV2 Model

MobileNetV2 is a streamlined deep convolutional neural network (CNN) model crafted explicitly for creating mobile phone applications focused on performing classification tasks [33]. The fundamental idea underlying the MobileNetV2 model revolves around creating connections between successive bottleneck layers. The architecture showcases an inverted residual design, incorporating 19 residual bottleneck layers, preceded by 32 total convolution layers. These convolution layers carry out depth-based convolutions, employing non-linear filter characteristics.

#### 2.4.2. ResNet50V2 Model

ResNet50V2 consists of units that propagate identities in both forward and backward directions, adhering to a residual nature [34]. The use of block-to-block propagation ensures the maintenance of high classification accuracy. The inclusion of these residual mappings significantly facilitates and generalizes the training process. In ImageNet or COC contests, ResNet models often surpass 100 layers, demonstrating exceptional accuracy.

#### 2.4.3. Xception Model

Xception adopts a straightforward modular structure based on Google’s Inception model [35]. It consists of three main blocks—entry, central, and exit—each equipped with distinct convolutional layers and ReLU activation functions. The input image size was set at 299 × 299 × 3. The entry flow processes the input, extracting features of dimensions 19 × 19 × 728. Residual connections ensure that the maximum value of each layer becomes the output after every block. In the central block of the feature map, the map remains unchanged despite undergoing convolution layers nine times. For a standard-size input image, the output of the final component comprised 2048 features. Ultimately, the prediction layer receives these features via a fully connected (FC) layer, and adjustments are made to the final layers to accommodate binary classification.

### 2.5. Experimental Setup

Our training and evaluation platform comprised the Kaggle environment and the powerful TensorFlow library. We deliberately chose three renowned pretrained CNN models—ResNet50V2, Xception, and MobileNetV2—based on their documented high accuracy in similar tasks [32]. To benefit from established best practices and maintain consistency, we chose hyperparameters that have demonstrated effectiveness in the above-mentioned model development research. This includes a batch size of 32; a maximum of 30 epochs; the utilization of the Adagrad optimizer; the ReLU activation function; a learning rate of 0.001; and the categorical cross-entropy loss function, which is suitable for binary classification tasks. These specific parameter values were carefully fine-tuned based on the findings from previous ablation studies, aimed at achieving optimal training performance for the selected algorithms.

**Figure 3:**
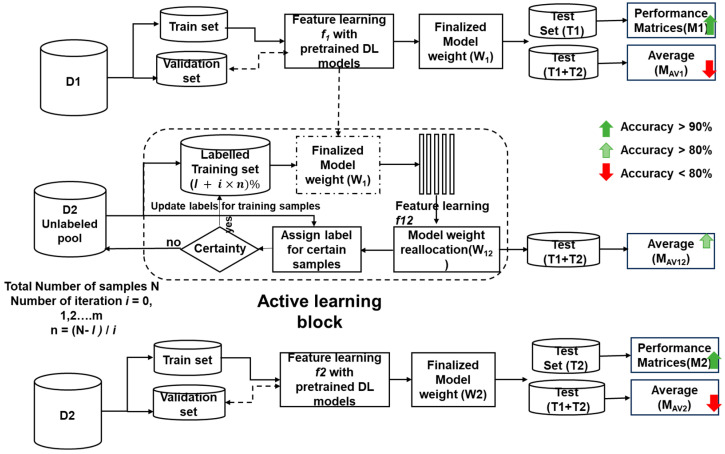
Domain adaptation with the active learning approach for ASD diagnosis process using facial images.

As illustrated in Figure 3, our methodology unfolds in three phases:

Phase 1: Initial Evaluation and Domain Adaptation Assessment

i. Train and evaluate all three models (ResNet50V2, Xception, MobileNetV2) on the D1 dataset;
ii. Extract the learned weights (*w*_1_) after initial feature learning (*f*_1_);
iii. Evaluate the combined test set (T1 + T2) using *w*_1_ weights to assess the initial extent of domain adaptation.

Phase 2: Active Learning for Enhanced Domain Adaptation

i. Initialize active learning with models containing *w*_1_ weights and limited labeled samples (*l*);
ii. Over *m* iterations,
  - Use the models to label unlabeled samples from D2, iteratively updating weights (*w*_12_);
  - Evaluate the performance of T2 against the current number of labeled samples.
iii. Train the models with the labeled D2 dataset (100%);
iv. Finally, evaluate the combined test set using these models to gauge the overall effectiveness of active learning in enhancing domain adaptation.

Phase 3: Final Comparison with ImageNet Pretraining

i. Retrain the ImageNet-pretrained models directly on the labeled D2 dataset;
ii. Extract the final learned weights (*w*_2_) after feature learning (*f*_2_);
iii. Evaluate the performance of T2 and the combined test set using *w*_2_ weights.

Regarding performance evaluation, a comprehensive range of metrics was utilized, which are accuracy, area under the curve (AUC), precision, recall, and f1-score, as described in Equations (6)–(9).

(6) $$\mathrm{Accuracy}=\frac{T_{p}+T_{n}}{T_{p}+T_{n}+F_{n}+F_{p}}$$

(7) $$\mathrm{Precision}=\frac{T_{p}}{T_{p}+F_{p}}$$

(8) $$\mathrm{recall}=\frac{T_{p}}{T_{p}+F_{n}}$$

(9) $$F1-\mathrm{score}=2\times \frac{Precision\times recall}{Precision+recall}$$

## 3. Results

For code development, we utilized the Python programming language [36], while the code was executed on the Kaggle platform [37]. After the completion of the model training, the obtained findings were analyzed using a range of data analysis tools, such as Matplotlib, sklearn, and Pandas. This study specifically emphasized three separate deep learning models, namely MobileNetV2, ResNet50V2, and Xception, and the chosen networks adhered to ideal hyperparameters and optimizer settings, as outlined in ablation research by Alam et al. [32]. The performance evaluation in this study involved measuring accuracy, precision, recall, and F1-score, which were computed using Equations (6)–(9).

### 3.1. Performance Evaluation after Transfer Learning with D1

Table 2 presents the evaluation results of the different performance parameters after receiving training on dataset D1 and later testing against T1 and the combined test set. Following transfer learning, the weight *w*_1_ was consistently employed across the three models trained on the Kaggle dataset. The highest level of accuracy, precisely 95%, was achieved using the Xception model, with 98% AUC while evaluating the performance metrics M_1_ on the test set T1. Upon performing an evaluation using a combined dataset that included test samples from both datasets, the average performance metrics of M_av1_ decreased to 73.5% for accuracy using ResNet50V2. The AUC was also reduced to 75.5% for the same model. All the performance data of M_1_ exhibited a consistent accuracy above 90%. However, there was a notable decrease, with M_av1_ exhibiting a decline to approximately a range of 68% to 73.5%. This decrease may be directly attributed to domain shift, as visually depicted in Figure 2.

### 3.2. Performance Evaluation after Transfer Learning with D2

In Table 3, a similar pattern is observed when assessing the performance of M_2_ with T2 after training with D2. The obtained results indicate the highest accuracy of around 96% and an AUC of 97% for ResNet50V2.

**Figure 4:**
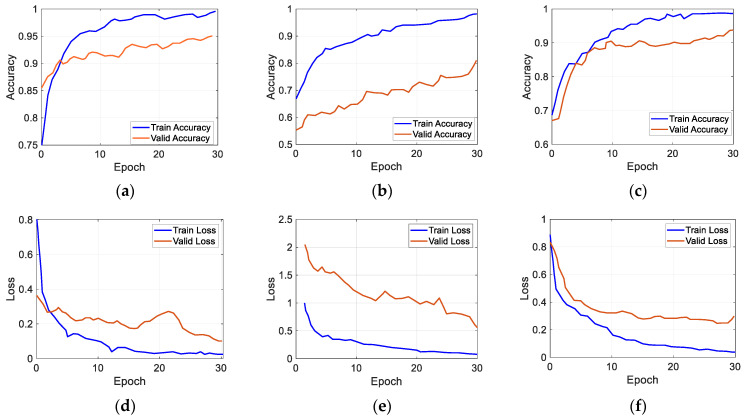
Graphical representations of training and validation accuracies of (**a**) ResNet50V2, (**b**) MobileNetV2, and (**c**) Xception model and training and validation losses of (**d**) ResNet50V2, (**e**) MobileNetV2, and (**f**) Xception model for face alignment.

The CNN models were trained using the identical technique and hyperparameter set outlined in the research conducted by Alam et al. (2022) [32]. The evaluation outcome, denoted as M_2_, also demonstrated promising performance for the Xception model, with an accuracy of 94% and an AUC (area under the curve) of 98%. However, they inaccurately predicted the features from different source datasets. The ResNet50V2 model achieved a M_av2_ accuracy of 75.9% and an AUC of 78% when tested on a mixed dataset of facial images from two domains. The training and validation accuracy plots are shown in Figure 4a–c for the ResNet50V2, MobileNetV2, and Xception models, respectively. Similarly, Figure 4d–f present the training and validation loss graphs according to the exact chronology.

### 3.3. Performance Evaluation after Active Learning Using D2

The weight, denoted as *w*_1_, was obtained by transfer learning using dataset D1 and subsequently employed in the active learning process. In the initial stage, the *l* number of labeled instances was utilized for feature learning, and later, the model weight was reassigned as *w*_12_.

**Figure 5:**
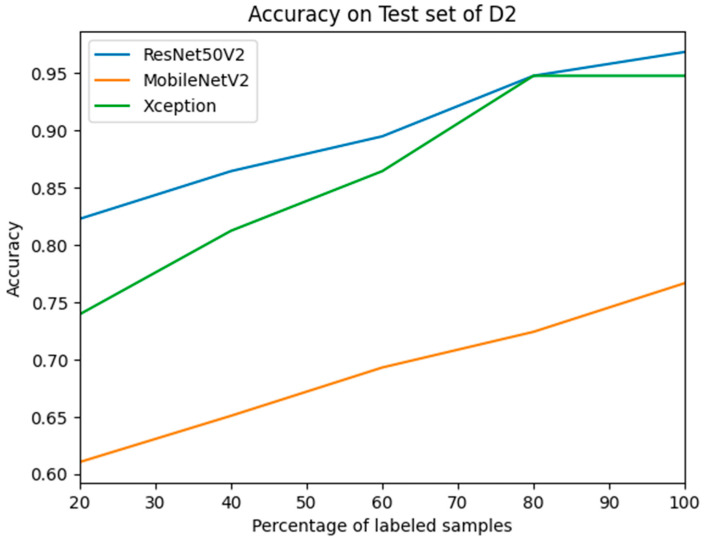
The trade-off between accuracy and the percentage of labeled samples during active learning using D2.

T2 and the combined dataset underwent evaluation using the model-weighted *w*_12_ during the assessment phase. After each iteration, labels were assigned to specific unlabeled samples based on the least confidence calculation. With each successive iteration, additional labeled samples were integrated, prompting the retraining of the models with an augmented quantity of labeled data. The samples displaying the lowest confidence levels were retained in the unlabeled pool for subsequent labeling in subsequent iterations. Figure 5 visually represents the assessment outcomes for T2, showcasing the evolution of labeled samples. Throughout the iterative training process of M_12_, a discernible trade-off between the number of labeled samples and accuracy was observed. As the quantity of labeled samples increased, accuracy exhibited an upward trajectory after each iteration. Notably, the accuracy of ResNet50V2 reached its pinnacle at 96.9% when the model weight *w*_1_ was updated following training with a completely labeled dataset D2, as detailed in Table 4. This signifies a marked improvement, surpassing the 96% accuracy achieved by weight *w*_2_ when solely trained with D2 using transfer learning. Furthermore, this accuracy was further enhanced through active learning, enriching the model’s learning process with features beyond those acquired solely through transfer learning. Upon evaluating the combined test set, a substantial increase of 80% in accuracy was observed for Xception and 79% for ResNet50V2, following training and reassigning the weight from *w*_1_ to *w*_12_ using the active learning method on D2, consisting of 100% labeled images. The discernible enhancement in performance is underscored by the area under the curve (AUC) for M_av12_, surpassing 80% for all models and reaching the highest value of 84.1% for ResNet50V2. This impressive outcome was achieved by updating the model through prior training on a dataset from a distinct domain featuring facial images belonging to the same classes.

## 4. Discussion

This study focused on the early diagnosis of autism using an optimized strategy that incorporates active learning based on domain adaptation. The study’s findings provide valuable insights, as demonstrated by the performance evaluation conducted during different phases of the experiment. The significance of domain adaptation is substantiated by investigating the samples from two distinct datasets, as illustrated in Figure 2. Current autism screening methods using facial images often face limitations when dealing with diverse data sources. Typically, models are trained on one specific dataset and tested on another from the same domain. Introducing a new dataset from a different domain challenges the model’s ability to generalize and accurately distinguish between autistic and normal control children. The discrepancy in domain-specific features, even for the same class, can lead to inaccurate predictions. Firstly, our proposed method can generalize the domain-specific features and differentiate between different classes more accurately, as the new weights have features from both domains after performing active learning. Secondly, this method also mitigates the annotation and labeling load, reducing the possibilities of human bias. Overall, this method is much more robust for prediction using new unknown data, as these data undergo training in both domains but require reduced manual work for the data preprocessing stages.

### 4.1. Evaluation of Same-Domain Test Sets

**Figure 6:**
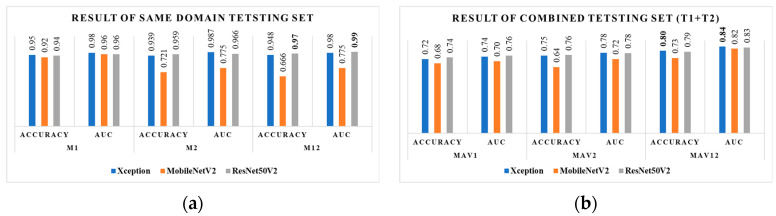
The plot of accuracy and AUC evaluated on (**a**) the same-domain test set and (**b**) a different-domain test set.

The conventional study demonstrates that the model accuracy remained consistently above 90% when both training and test datasets were sourced from the same domain. This emphasizes the importance of domain-specific adaptation for reliable autism diagnosis. Within the transfer learning framework, we trained three models (ResNet50V2, Xception, and MobileNetV2) on dataset D1 and tested them on T1. Xception emerged as the top performer with 95% accuracy, showcasing its capacity to adapt to a similar domain. This trend continued when evaluating T2 using transfer learning from D2. ResNet50V2 achieved the highest accuracy at 96%, accompanied by an impressive AUC of 97%. These results confirm the effectiveness of transfer learning in improving model performance for closely related domains. Interestingly, our study further reveals that active learning surpasses even the impressive performance of transfer learning. By employing active learning with ResNet50V2 and updating its weights (w_1_ to w_12_) during training on fully labeled dataset D2, we observed a peak accuracy of 96.9%. This exceeds the accuracy achieved through transfer learning with D2 alone, as illustrated in Figure 6. This finding highlights the significant potential of active learning in enhancing model generalizability and accuracy across diverse datasets. Our study highlights the importance of considering domain specificity and employing robust techniques like transfer learning and active learning to improve the accuracy and generalizability of autism screening models based on facial images. Further research should explore these techniques across broader and more diverse datasets, paving the way for more reliable and universally applicable tools for early autism diagnosis. 

### 4.2. Evaluation of Different-Domain Test Sets

Nevertheless, while assessing the combined test dataset T1 + T2 after being trained with D1, it was seen that the average accuracy experienced a drop to 73.5%. Additionally, ResNet50V2 exhibited a decrease in AUC to 75.5%. As anticipated, the evaluation metric M_av2_ exhibited a drop in the combined test set due to the domain change, which was trained on D2 only. This domain shift resulted in inaccuracies in predicting features from different source datasets. After the implementation of active learning, the assessment of the combined test set demonstrated significant enhancements, as depicted in Figure 6b. The Xception and ResNet50V2 models improved accuracy by 80% and 79%, respectively. All models had an AUC exceeding 80%, with ResNet50V2 achieving the highest result of 84.1%. The improvement was attained by updating the model through pretraining on a dataset, D1, followed by the active learning approach utilizing a different-domain dataset, D2.

### 4.3. Explaining AI in Active Learning Context

Explainable AI refers to the ability of an AI system to clearly communicate its logic and the methods it uses to make decisions in a way that humans can comprehend [38]. Active learning refers to the ability of an AI system to label samples and learn features simultaneously, as opposed to other methods. The connection between these two notions is rooted in the fact that explainability can potentially augment the efficacy of active learning by offering valuable insights into the decision-making process of the AI system. One method to clarify the decision-making process of a neural network is by employing visualization tools such as Grad-CAM [39]. This technique identifies crucial areas inside an input image that significantly impact the neural network’s prediction. By visualizing the learned knowledge, we can better understand where and how to concentrate on facial images. This helps identify and fix issues in active learning models and provides evidence of their superiority in transfer learning approaches.

**Figure 7:**
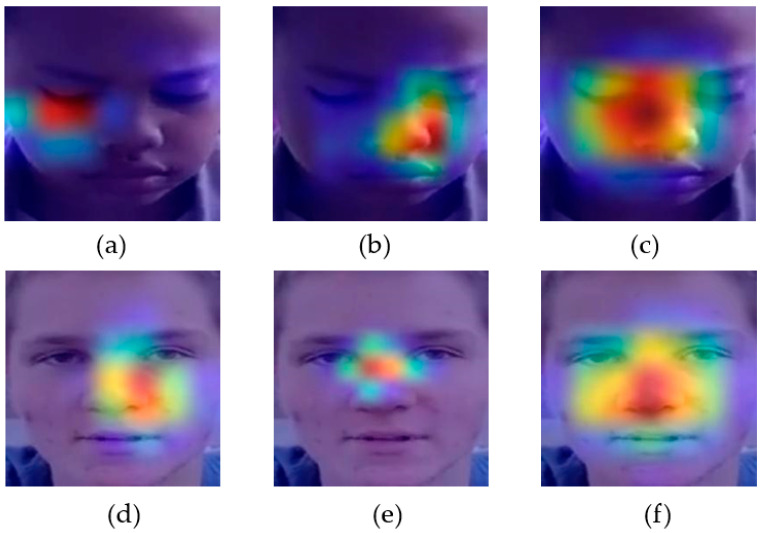
Grad-CAM representation of a random sample of T2 (**a**) misclassified using *w*_1_, (**b**) rightly predicted using *w*_2_, (**c**) rightly predicted using *w*_12_ (active learning), (**d**) misclassified using *w*_1_, (**e**) misclassified using *w*_2_, and (**f**) predicted using *w*_12_ (active learning) for the Xception model.

Figure 7 displays the Grad-CAM feature maps for two randomly selected ASD samples from the TYUIA test set T2 using the Xception model. Figure 7a depicts the first sample, which, when assessed using the weight *w*_1_ (different domain), incorrectly classified the sample as NC due to the model’s failure to consider the specific facial landmarks accurately. According to previous research [40], the Xception model should primarily concentrate on the eye and nose region. The model accurately predicted the sample using the weight *w*_2_, which belonged to the same domain as the model trained with D2, as shown in Figure 7b. The use of active learning resulted in domain adaptation. Figure 7c demonstrates that when combined with active learning techniques, the Grad-CAM feature map effectively focuses on specific facial features, resulting in an accurate prediction. The model incorrectly predicted the subsequent example with the weight *w*_1_, as depicted in Figure 7d. Due to the lack of notable attention to the facial landmarks, the test sample was misclassified, even though the model’s weight, *w*_2_, was in the same domain as that shown in Figure 7e. In such circumstances, active learning emphasizes facial landmarks and facilitates accurate prediction for the autistic population, as Figure 7f depicts.

### 4.4. Comparative Insights: Benchmarking against Recent Research Studies

Incorporating facial images in ASD screening is a relatively new development, as indicated by the scarcity of previous research on the topic. Thus, we focused on two separate datasets for diagnosing autism spectrum disorder (ASD): (fMRI) based on neuroimaging and facial image datasets. Considering this, we investigated the domain adaptation tasks and their corresponding accuracy for both types of datasets, as shown in Table 5.

Numerous studies have leveraged functional magnetic resonance imaging (fMRI) data to diagnose autism spectrum disorder (ASD), notably employing the ABIDE dataset collected from diverse locations. Noteworthy contributions by Kunda et al. (2023) [21], Wang et al. (2020) [41], Jiang et al. (2019) [42], and Bhaumik et al. (2018) [43] utilized varied methodologies such as MIDA, maLRR, MCDA, and PLS, respectively, with documented maximum accuracies ranging from 62% to 73.45%. The inherent challenges of domain adaptation within neuroimaging datasets become evident due to the ABIDE dataset’s heterogeneity, originating from various sources. While the application of facial images in ASD diagnosis has received comparatively less attention, recent endeavors have yielded promising outcomes. For instance, Lu et al. (2021) [44] utilized the Federation dataset to analyze facial images from Kaggle ASD and East Asian datasets, achieving an accuracy rate of 75.2%. In our proposed approach, we harnessed facial photos from the Kaggle ASD dataset and a novel YTUIA dataset. Employing active learning, we achieved a noteworthy accuracy rate of 80.01% on a combined test set, surpassing the documented accuracy levels in previous neuroimaging studies. This underscores the potential significance of exploring these datasets for ASD screening. The substantial accuracy level attained with our proposed approach emphasizes the distinct domain adaptation challenges between neuroimaging and facial image datasets, underscoring the imperative need for tailored methodologies. The limited research on face images in ASD screening underlines the necessity of further exploration in this domain. Encouraging findings suggest that face images could be a valuable and easily accessible tool for diagnosing ASD, offering an alternative or complementary approach to conventional neuroimaging techniques.

Despite the significant advancements achieved, it is important to acknowledge that this study is not without its limitations, which warrant careful consideration. One such challenge lies in the observed decline in performance when the model was tested on the preceding domain, highlighting the need for further optimization and refinement of the approach. Moreover, fluctuations in labeling accuracy during the active learning stages pose additional hurdles, requiring meticulous monitoring and adjustment throughout the process. Furthermore, the dataset utilized in this study lacks rigorous clinical validation and comprehensive demographic information, which introduces potential biases and limitations in generalizing the results. Therefore, it is essential to approach the interpretation of findings with caution and recognize the need for additional validation and refinement in future research endeavors.

## 5. Conclusions

This study leverages the powerful combination of active learning and domain adaptation to optimize early ASD, introducing a novel approach in a domain characterized by diverse datasets. While traditional facial image screening for autism typically remains within the same domain, achieving consistent accuracy above 90%, our methodology breaks new ground. Xception attained a peak accuracy of 95%, and ResNet50V2 achieved an impressive 96%, demonstrating the effectiveness of transfer learning on the Kaggle ASD and TYUIA datasets. Active learning contributed to a significant accuracy improvement, by 2%, on the same-domain test set. However, when faced with a combined test set amalgamating datasets from distinct domains, there was a marginal decrease in average accuracy from 73.5% to 75.5%, highlighting challenges, particularly for ResNet50V2 due to domain shift. Yet, active learning resulted in outstanding improvements, with Xception and ResNet50V2 achieving accuracy enhancements of 80% and 79%, respectively. All models consistently achieved over 80% AUC, underscoring the robustness of our innovative methodology. Pretraining on Kaggle ASD and subsequent active learning with TYUIA substantiate the efficacy of our approach. Despite these advancements, the study is not without limitations. The decline in performance when tested on the preceding domain and variability in labeling accuracy during active learning stages present challenges. Additionally, the dataset lacks rigorous clinical validation and comprehensive demographic information, necessitating caution in generalizing results. In conclusion, while our proposed methodology faces hurdles in domain shift complications, the findings showcase its potential for early autism detection. Future work should concentrate on developing a domain adapter, incorporating both model weight and features, and testing it on a clinically validated dataset to assess its effectiveness in mitigating domain shift challenges.

## Figures and Tables

**Table 1:** Comparative performance of facial image screening algorithms for autism diagnosis.

Refs.	Author	Algorithm	Accuracy	Dataset	Active Learning	Domain Adaptation
[16]	M. Derbali et al. (2023)	VGGFace	92.30	Kaggle	No	NR
[17]	A. Mouatasim et al. (2023)	Densnet121	91.00	Kaggle	No	NR
[18]	L. K. Gaddala et al. (2023)	VGG16	88.00	Kaggle	No	NR
[19]	N. Kaur et al. (2023)	VGG16	68.54	Kaggle	No	NR
[20]	A. Singh et al. (2023)	MobileNet	88.00	Kaggle	No	NR

NR = not required.

**Table 2:** The performance of transfer learning on the Kaggle dataset.

	M_1_ = Evaluation on T1 with Weight *w*_1_				M_av1_ = Evaluation on T1 + T2 with Weight *w*_1_			
Model	Accuracy	Precision	f1-Score	AUC	Accuracy	Precision	f1-Score	AUC
Xception	0.950	0.950	0.940	0.98	0.720	0.720	0.720	0.743
MobileNetV2	0.920	0.920	0.920	0.96	0.680	0.680	0.680	0.699
ResNet50V2	0.940	0.940	0.940	0.96	0.735	0.735	0.735	0.755

**Table 3:** The performance of transfer learning on the TYUIA dataset.

	M_2_ = Evaluation on T2 with Weight *w*_2_				M_av2_ = Evaluation on T1 + T2 with Weight *w*_2_			
Model	Accuracy	Precision	f1-Score	AUC	Accuracy	Precision	f1-Score	AUC
Xception	0.939	0.939	0.939	0.987	0.754	0.754	0.754	0.780
MobileNetV2	0.721	0.721	0.721	0.775	0.641	0.641	0.641	0.720
ResNet50V2	0.959	0.959	0.959	0.966	0.759	0.759	0.759	0.778

**Table 4:** The performance of transfer learning on the TYUIA dataset.

	M_12_ = Evaluation on T2 with Weight *w*_12_				M_av12_ = Evaluation on T1 + T2 with Weight *w*_12_			
Model	Accuracy	Precision	f1-Score	AUC	Accuracy	Precision	f1-Score	AUC
Xception	0.948	0.948	0.948	0.978	0.801	0.801	0.801	0.841
MobileNetV2	0.666	0.667	0.667	0.775	0.731	0.731	0.731	0.820
ResNet50V2	0.969	0.969	0.969	0.991	0.790	0.790	0.790	0.833

**Table 5:** Comparison of performance parameters with recent research.

Detail of Dataset	Method	Accuracy	Refs.
Neuroimaging dataset (fMRI)			
ABIDE from 20 different sites	MIDA	73.00	[21]
ABIDE from 5 different sites	maLRR	73.44	[41]
ABIDE from 17 different sites	MCDA	73.45	[42]
ABIDE	PLS	62.00	[43]
Facial image dataset			
1.Kaggle ASD, East Asian	Federated learning	75.20	[44]
1.Kaggle ASD, 2. TYUIA	Active learning	80.01	Proposed

## Data Availability

The data and images utilized in this research were obtained from publicly available online sources. No human subjects were involved in the study, and all data were handled in accordance with ethical standards. Kaggle Dataset link: https://github.com/mm909/Kaggle-Autism (accessed on 5 March 2024). YTUIA Dataset link: https://drive.google.com/drive/u/0/folders/1g8iyO2Q0jnWLj6w6l5Nb8vm7GogTzK0J?fbclid=IwAR3efHMw69tkauKKGOZVwOMXXdYY2pqKPK__Cl621FSh6IoLJM02dVhffJ4 (accessed on 5 March 2024).
